# Assessment of Brain Magnetic Resonance and Spectroscopy Imaging Findings and Outcomes After Pediatric Cardiac Arrest

**DOI:** 10.1001/jamanetworkopen.2023.20713

**Published:** 2023-06-30

**Authors:** Ericka L. Fink, Patrick M. Kochanek, Sue R. Beers, Robert R. S. B. Clark, Rachel P. Berger, Hülya Bayir, Alexis A. Topjian, Christopher Newth, Craig Press, Aline B. Maddux, Frederick Willyerd, Elizabeth A. Hunt, Ashley Siems, Melissa G. Chung, Lincoln Smith, Leslie Doughty, J. Wesley Diddle, Jason Patregnani, Juan Piantino, Karen Hallermeier Walson, Binod Balakrishnan, Michael T. Meyer, Stuart Friess, Jose Pineda, David Maloney, Pamela Rubin, Tamara L. Haller, Amery Treble-Barna, Chunyan Wang, Vince Lee, Jessica L. Wisnowski, Subramanian Subramanian, Srikala Narayanan, Stefan Blüml, Anthony Fabio, Ashok Panigrahy, and the POCCA Investigators

**Affiliations:** 1 Department of Critical Care Medicine, Division of Pediatric Critical Care Medicine, University of Pittsburgh Medical Center, Children’s Hospital of Pittsburgh, Pittsburgh, Pennsylvania; 2 Department of Pediatrics, University of Pittsburgh Medical Center, Children’s Hospital of Pittsburgh, Pittsburgh, Pennsylvania; 3 Safar Center for Resuscitation Research, University of Pittsburgh Medical Center, Pittsburgh, Pennsylvania; 4 Department of Psychiatry, University of Pittsburgh School of Medicine, Pittsburgh, Pennsylvania; 5 Department of Anesthesia and Critical Care Medicine, Children’s Hospital of Philadelphia, University of Pennsylvania Perelman School of Medicine, Philadelphia; 6 Department of Anesthesiology and Critical Care Medicine, Children’s Hospital of Los Angeles, Los Angeles, California; 7 Department of Pediatrics, University of Colorado School of Medicine and Children’s Hospital Colorado, Aurora; 8 Department of Pediatrics, Phoenix Children’s Hospital, Phoenix, Arizona; 9 Departments of Anesthesiology and Critical Care Medicine, and Pediatrics, Johns Hopkins Children’s Center, Baltimore, Maryland; 10 Department of Pediatrics, Division of Critical Care Medicine, and Pediatric Neurology, Nationwide Children’s Hospital, Columbus, Ohio; 11 Department of Pediatrics, University of Washington School of Medicine, Seattle; 12 Department of Pediatrics, Cincinnati Children’s Hospital Medical Center, Cincinnati, Ohio; 13 Department of Pediatrics, Children’s National Medical Center, Washington, DC; 14 Department of Pediatrics, Oregon Health & Science University, Portland; 15 Department of Pediatrics, Children’s Healthcare of Atlanta, Atlanta, Georgia; 16 Department of Pediatrics, University of Wisconsin School of Medicine and Public Health, Madison; 17 Department of Pediatrics, St Louis Children’s Hospital, St Louis, Missouri; 18 Department of Anesthesia Critical Care, Mattel Children’s Hospital, University of California, Los Angeles; 19 Department of Epidemiology, University of Pittsburgh School of Medicine, Pittsburgh, Pennsylvania; 20 Department of Physical Medicine and Rehabilitation, University of Pittsburgh School of Medicine, Pittsburgh, Pennsylvania; 21 Department of Radiology, University of Pittsburgh Medical Center, Children’s Hospital of Pittsburgh, Pittsburgh, Pennsylvania; 22 Department of Radiology, Children’s Hospital of Los Angeles, Los Angeles, California

## Abstract

**Importance:**

Morbidity and mortality after pediatric cardiac arrest are chiefly due to hypoxic-ischemic brain injury. Brain features seen on magnetic resonance imaging (MRI) and magnetic resonance spectroscopy (MRS) after arrest may identify injury and aid in outcome assessments.

**Objective:**

To analyze the association of brain lesions seen on T2-weighted MRI and diffusion-weighted imaging and N-acetylaspartate (NAA) and lactate concentrations seen on MRS with 1-year outcomes after pediatric cardiac arrest.

**Design, Setting, and Participants:**

This multicenter cohort study took place in pediatric intensive care units at 14 US hospitals between May 16, 2017, and August 19, 2020. Children aged 48 hours to 17 years who were resuscitated from in-hospital or out-of-hospital cardiac arrest and who had a clinical brain MRI or MRS performed within 14 days postarrest were included in the study. Data were analyzed from January 2022 to February 2023.

**Exposure:**

Brain MRI or MRS.

**Main Outcomes and Measures:**

The primary outcome was an unfavorable outcome (either death or survival with a Vineland Adaptive Behavior Scales, Third Edition, score of <70) at 1 year after cardiac arrest. MRI brain lesions were scored according to region and severity (0 = none, 1 = mild, 2 = moderate, 3 = severe) by 2 blinded pediatric neuroradiologists. MRI Injury Score was a sum of T2-weighted and diffusion-weighted imaging lesions in gray and white matter (maximum score, 34). MRS lactate and NAA concentrations in the basal ganglia, thalamus, and occipital-parietal white and gray matter were quantified. Logistic regression was performed to determine the association of MRI and MRS features with patient outcomes.

**Results:**

A total of 98 children, including 66 children who underwent brain MRI (median [IQR] age, 1.0 [0.0-3.0] years; 28 girls [42.4%]; 46 White children [69.7%]) and 32 children who underwent brain MRS (median [IQR] age, 1.0 [0.0-9.5] years; 13 girls [40.6%]; 21 White children [65.6%]) were included in the study. In the MRI group, 23 children (34.8%) had an unfavorable outcome, and in the MRS group, 12 children (37.5%) had an unfavorable outcome. MRI Injury Scores were higher among children with an unfavorable outcome (median [IQR] score, 22 [7-32]) than children with a favorable outcome (median [IQR] score, 1 [0-8]). Increased lactate and decreased NAA in all 4 regions of interest were associated with an unfavorable outcome. In a multivariable logistic regression adjusted for clinical characteristics, increased MRI Injury Score (odds ratio, 1.12; 95% CI, 1.04-1.20) was associated with an unfavorable outcome.

**Conclusions and Relevance:**

In this cohort study of children with cardiac arrest, brain features seen on MRI and MRS performed within 2 weeks after arrest were associated with 1-year outcomes, suggesting the utility of these imaging modalities to identify injury and assess outcomes.

## Key Points

**Question:**

Are regional brain features assessed with magnetic resonance imaging (MRI) and magnetic resonance spectroscopy (MRS) within 2 weeks of pediatric cardiac arrest associated with outcomes at 1 year?

**Findings:**

In this multicenter cohort study of 98 children with cardiac arrest who underwent clinical MRI or MRS, higher MRI Injury Score, increased lactate, and decreased N-acetylaspartate in regions of interest were associated with an unfavorable outcome at 1 year.

**Meaning:**

These findings suggest that brain imaging using MRI or MRS during the first 2 weeks after pediatric cardiac arrest may aid in outcome assessment.

## Teaser

This cohort study analyzes the association of brain lesions seen on magnetic resonance imaging and concentrations of lactate and N-acetylaspartate seen on magnetic resonance spectroscopy with 1-year outcomes after pediatric cardiac arrest.

## Introduction

Children resuscitated from cardiac arrest have high morbidity and mortality rates, mostly due to global cerebral hypoxic-ischemic injury.^1,2^ Accurate tools to assess patient outcomes are essential for shared clinical decision-making. Consensus statements for postarrest care of children include brain computed tomography or magnetic resonance imaging (MRI) to assess outcomes, but high-quality evidence is lacking.^3,4,5^

Conventional T2-weighted imaging and diffusion-weighted imaging (DWI) sequences can detect injury within hours to days after a hypoxic-ischemic event. Single-center studies^6,7,8^ of pediatric cardiac arrest have found an association of regional brain injury seen on T2-weighted imaging and DWI sequences and outcomes. Furthermore, studies^8,9^ using magnetic resonance spectroscopy (MRS), which analyzes brain biochemistry, show that increased lactate and decreased N-acetylaspartate (NAA) are promising biomarkers associated with outcomes after pediatric cardiac arrest. A reduction of NAA is associated with neuronal and axonal injury, whereas lactate accumulates when pyruvate oxidation in the citric acid cycle is impaired.^10^ However, multicenter, prospective studies of children with cardiac arrest are lacking.

The objective of this prospective multicenter cohort study was to analyze the association of brain features seen on MRI and MRS performed within 2 weeks among children who experienced cardiac arrest with the composite outcome of death or unfavorable adaptive behavior at 1 year. We hypothesized that brain MRI and MRS features would be associated with the composite outcome.

## Methods

### Study Design and Setting

The Personalizing Outcomes After Child Cardiac Arrest (POCCA) prospective cohort study^12^ was performed at 14 pediatric intensive care units in the US between May 16, 2017, and August 19, 2020, including follow-up through 1 year. The University of Pittsburgh institutional review board approved the study centrally. Independent institutional review board approval was obtained by 2 sites (Children’s Healthcare of Atlanta and Children’s Wisconsin), because they could not participate in the central review board. Written informed consent from a parent or guardian was required for participation and written and/or verbal assent was obtained from the children when appropriate according to local center guidelines. This study followed the Strengthening the Reporting of Observational Studies in Epidemiology (STROBE) reporting guideline for cohort studies.^11^

### Participants

Eligible children included those aged 48 hours to 17 years who were enrolled in the parent study^12^ and underwent a brain MRI or MRS as part of routine clinical care within the first 14 days after being resuscitated from in-hospital or out-of-hospital cardiac arrest. See eFigure 1 in Supplement 1 for more information on participant eligibility.

### Data Collection

Cardiac arrest, resuscitation, and postrestoration of spontaneous circulation data were collected from the medical record using a standardized case report form.^13,14^ Patient demographics, including race and ethnicity, were extracted from the medical record using National Institutes of Health–recommended categories, and included African American or Black, Asian, White, and unknown race, and Hispanic ethnicity.^14^ Race and ethnicity were recorded to evaluate for outcome differences.^15^

### Brain MRI and MRS

Brain MRI was performed as routine clinical care (eTable 1 in Supplement 1). Brain MRS was performed as either routine clinical care or as research if the center did not routinely perform MRS for this indication. MRI safety screenings were performed using local procedures. For patients who underwent MRI or MRS multiple times, we included only the first MRI or MRS sequence for this analysis.

MRI and MRS procedures and protocols for data acquisition, image extraction, and anonymization for deidentified data sharing were distributed to centers (eAppendix 1 in Supplement 1). Centers collaborated with a pediatric neuroradiologist, MR technician, and MR physicist to provide information on local MRI capabilities before enrollment and had individual training sessions with study personnel. MRI data were acquired on any qualified MR scanner with MRS metabolites analyzed only if performed on a 3-Tesla scanner. Deidentified brain MRI and MRS images were uploaded to the study cloud (Ambra Health [now Intelerad]).

The MRI protocol included axial and coronal T2-weighted sequences. Whole-brain DWI was performed at b = 1000 seconds/mm^2^ with 6 directions. In-plane resolution was 1.1 × 1.1 × 6.0 mm^3^ with no gap. Scan time was echo time to repetition time of 87.4 per 10 000 milliseconds.

Brain MRI images were scored and analyzed by 2 pediatric neuroradiologists (S.N. and S.S.) who were blinded to both patient outcome and each other’s scores. T2-weighted and DWI images were examined for signal intensity abnormalities and scored using a system adapted from Hirsch et al^16^ (eAppendix 2 in Supplement 1). Findings in white and gray matter were reported separately when appropriate. Lesions seen on T2-weighted imaging and DWI were also scored by severity (0 = none, 1 = mild, 2 = moderate, 3 = severe). MRI Injury Score was a sum of T2-weighted imaging and DWI lesions in gray and white matter (maximum score, 34). The Cortex Score was a sum of gray and white matter scores from 4 cortical lobes. The Deep Gray Matter Score was a sum of basal ganglia (lenticular and caudate) and thalamus scores. Interobserver variability was described, and disagreement was resolved by consensus.

MRS data were acquired using a single-voxel point-resolved spectroscopy sequence with an echo time of 35 milliseconds and repetition time of 2 seconds (3-Tesla). MRS data were obtained from 4 regions of interest (ROIs): (1) basal ganglia, (2) thalamus, (3) parietal white matter, and (4) parietooccipital gray matter (eAppendix 3 in Supplement 1). These ROIs correspond to those regions most vulnerable to global hypoxic-ischemic injury and were standardized in the protocol.^8,17,18^ Axial, sagittal, and coronal orientations were assessed for accurate voxel placement. Volumes of ROIs were typically 3 to 6 cm^3^, and acquisition times (128 averages) were 3 to 5 minutes per ROI (eAppendix 1 in Supplement 1).

Raw MRS data files were transferred offline for fully automated postprocessing using LCModel software version 6.3-1P (Stephen Provencher Inc).^16,19^ LCModel processing provides objective measures for the signal-to-noise ratio and the spectral line width (full width at half maximum) for objective quality assessment. Spectra with a low signal-to-noise ratio of more than 2 SDs below the mean were discarded. We also excluded spectra with poor line width more than 2 SDs above the mean. From the remaining spectra, absolute concentrations (institutional units) of NAA and lactate were determined using the unsuppressed water signal as an internal reference, with a water content of 75% set as an approximation for our patient population. We also computed and analyzed the lactate-to-NAA ratio. Pediatric neuroradiologists verified MRS ROI placements at each center. MRS measurements were performed on 1 hemisphere only because cardiac arrest produces global hypoxia-ischemia.

### Outcomes

The primary outcome was the association of brain MRI Injury Score and MRS features with an unfavorable outcome (either death or survival with a Vineland Adaptive Behavior Scales, Third Edition^20^ [VABS-3] score <70) at 1 year. Patient follow-up was standardized and performed at each center as per the parent study.^12,20^

### Statistical Analysis

Frequencies and percentages were reported for categorical variables. Results were nonparametric and thus presented as median (IQR). Outcome group comparisons were made using Kruskal-Wallis, Mann-Whitney, Fisher exact, and χ^2^ tests, as appropriate. Simple and weighted κ statistics are reported for MRI scores (eTable 2 in Supplement 1).* P* < .05 was considered significant for unfavorable vs favorable outcomes at 1 year.

Multivariable logistic regression modeling was performed to evaluate the association of MRI Injury Score with 1-year outcomes. Variables initially tested in univariate models included MRI Injury Score, cause of arrest, arrest location, total number of epinephrine doses, first monitored rhythm, age, sex, and witnessed event status.^21,22,23^ Covariates significant at *P* < .20 were then evaluated using multivariable logistic regression models with stepwise selection and entry and removal levels of .20. A multivariable regression for MRS results was not possible due to limited sample size. Multivariable analysis of the area under the receiver operator characteristic curve (AUROC) was used to assess the accuracy of clinical variables alone and with MRI Injury Score to assess 1-year outcomes. Missing data were not imputed. Only patients with primary outcome data available were analyzed. Analyses were conducted using SAS statistical software version 9.2 (SAS Institute, Inc). Data analysis was conducted from January 2022 to February 2023.

## Results

### Participants

A total of 98 children were included in the study: 66 children underwent MRI (median [IQR] age, 1.0 [0.0-3.0] years; 28 girls [42.4%]; 46 White children [69.7%]), and 32 children underwent MRS (median [IQR] age, 1.0 [0.0-9.5] years; 13 girls [40.6%]; 21 White children [65.6%]) (Table 1). In the MRI group, 23 children (34.8%) had an unfavorable outcome and in the MRS group 12 children (37.5%) had an unfavorable outcome. Patient characteristics were not different among outcome groups. Among children who underwent MRI and had an unfavorable outcome, more cardiac arrest events were unwitnessed (13 events [56.5%]) and occurred out-of-hospital (20 events [87.0%]) compared with the 43 children who underwent MRI and had a favorable outcome (4 unwitnessed events [9.3%]; 18 out-of-hospital events [41.9%]; The cause of death was brain death declaration in 6 patients (31.6%) who underwent MRI (Table 2).

**Table 1.** Patient and Cardiac Arrest Characteristics for Children Who Underwent Brain MRI and MRS by Outcome Status at 1 Year^a^

Characteristic	Children who underwent MRI, No. (%)				Children who underwent MRS, No. %				
	Overall (N = 66)	Favorable outcome (n = 43)	Unfavorable outcome (n = 23)	*P* value^b^	Overall (N = 32)		Favorable outcome (n = 20)	Unfavorable outcome (n = 12)	*P* value^b^
Age, median (IQR), y	1.0 (0.0-3.0)	1.0 (0.0-8.0)	1.0 (0.0-2.0)	.50	1.0 (0.0-9.5)		1.0 (0.0-9.5)	0.5 (0.0-7.0)	.76
Sex									
Female	28 (42.4)	19 (44.2)	9 (39.1)	.80	13 (40.6)		8 (40.0)	5 (41.7)	>.99
Male	38 (57.6)	24 (55.8)	14 (60.9)		19 (59.4)		12 (60.0)	7 (58.3)	
Race									
African American or Black	7 (10.6)	6 (14.0)	1 (4.4)	.10	4 (12.5)		3 (15.0)	1 (8.3)	.06
Asian	3 (4.6)	1 (2.3)	2 (8.7)		1 (3.1)		0	1 (8.3)	
White	46 (69.7)	27 (62.8)	19 (82.6)		21 (65.6)		11 (55.0)	10 (83.3)	
Unknown	10 (15.2)	9 (20.9)	1 (4.4)		6 (18.8)		6 (30.0)	0	
Hispanic ethnicity^c^	7 (11.3) (n = 62)	5 (12.8) (n = 39)	2 (8.7) (n = 23)	>.99	3 (10.3) (n = 29)		3 (17.7) (n = 17)	0 (n = 12)	.25
Preexisting conditions^c^	39 (62.9) (n = 62)	26 (66.7) (n = 39)	13 (56.5) (n = 23)	.59	19 (61.3) (n = 31)		12 (63.2) (n = 19)	7 (58.3) (n = 12)	>.99
Primary cause of event^c^	n = 56	n = 40	n = 16		n = 24		n = 17	n = 7	
Asphyxia	40 (71.4)	26 (65.0)	14 (87.5)	.11	18 (75.0)		11 (64.7)	7 (100.0)	.13
Cardiac arrest	16 (28.6)	14 (35.0)	2 (12.5)		6 (25.0)		6 (35.3)	0	
Event location									
Out-of-hospital	38 (57.8)	18 (41.9)	20 (87.0)	<.001	21 (65.6)		10 (50.0)	11 (91.7)	.02
In-hospital	28 (42.4)	25 (58.1)	3 (13.0)		11 (34.4)		10 (50.0)	1 (8.3)	
Duration of cardiopulmonary resuscitation, median (IQR), min^c^	11.0 (5.0-23.5) (n = 56)	6.0 (4.0-16.0) (n = 38)	20.5 (8.0-40.0) (n = 18)	.002	11.0 (5.0-26.0) (n = 28)		5.5 (4.0-16.0) (n = 18)	21.0 (14.0-40.0) (n = 10)	.01
Epinephrine doses, median (IQR), No.^c^	2.0 (1.0-4.0) (n = 56)	2.0 (1.0-4.0) (n = 37)	2.0 (1.0-4.0) (n = 19)	.99	2.0 (1.0-3.0) (n = 27)		1.0 (2.0-3.0) (n = 16)	2.0 (1.0-4.0) (n = 11)	.25
Defibrillated^c^	10 (17.2) (n = 58	8 (21.1) (n = 38)	2 (10.0) (n = 20)	.47	6 (23.1) (n = 26)		5 (31.3) (n = 16)	1 (10.0) (n = 10)	.35
First monitored rhythm^c^	n = 56	n = 37	n = 19		n = 26		n = 17	n = 9	
Sinus bradycardia	21 (37.5)	16 (43.2)	5 (26.3)	.08	8 (30.8)		6 (35.3)	2 (22.2)	.33
Pulseless electrical activity	11 (19.6)	6 (16.2)	5 (26.3)		5 (19.2)		2 (11.8)	3 (33.3)	
Asystole	12 (21.4)	5 (13.5)	7 (36.8)		8 (30.8)		4 (23.5)	4 (44.4)	
Ventricular tachycardia or fibrillation	8 (14.3)	7 (18.9)	1 (5.3)		4 (15.4)		4 (23.5)	0	
Other (eg, normal sinus, sinus tachycardia)	4 (7.2)	3 (8.1)	1 (5.3)		1 (3.9)		1 (5.9)	0	
Witnessed cardiac event	49.0 (74.2)	39 (90.7)	10 (43.5)	<.001	19 (59.4)	16 (80.0)		3 (25.0)	.004
Bystander resuscitation									
Healthcare personnel	46 (69.7)	32 (74.4)	14 (60.9)	.28	19 (59.4)	13 (65.0)		6 (50.0)	.47
Nonhealth care personnel	20 (30.3)	11 (25.6)	9 (39.1)		13 (40.6)	7 (35.0)		6 (50.0)	

Abbreviations: MRI, magnetic resonance imaging; MRS, magnetic resonance spectroscopy.

^a^ A favorable outcome was defined as a Vineland Adaptive Behavioral Scale-3 (VABS-3) score of 70 or higher, and an unfavorable outcome was defined as a VABS score less than 70 or patient death.

^b^ *P* values are based on χ^2^ test for categorical variables and a Kruskal-Wallis test was used for medians.

^c^ Numbers in these rows differ from column totals because data were not available for some patients.

**Table 2.** Hospital-Based Therapies and Outcomes for Children With Brain Magnetic Resonance Imaging (MRI) and Magnetic Resonance Spectroscopy (MRS) by Outcome Status at 1 Year^a^

Characteristic	Children with MRI, No. (%)				Children with MRS, No. (%)			*P* value^b^
	Overall (N = 66)	Favorable outcome (N = 43)	Unfavorable outcome (N = 23)	*P* value^b^	Overall (N = 32)	Favorable outcome (N = 20)	Unfavorable outcome (N = 12)	
Time from cardiac arrest to imaging, median (IQR)d	5.0 (2.0-9.0)	5.0 (3.0-12.0)	2.0 (2.0-4.0)	<.001	5.0 (3.5-9.5)	7.5 (5.0-13.0)	4.0 (2.0-5.5)	.02
Hospital length of stay, median (IQR)d	21 (12-43)	28 (15-51)	12 (6-25)	.01	22 (12-44)	29 (15-52)	10 (5-24)	.01
Intensive care unit length of stay, median (IQR) days^c^	16 (9-27) (n = 65)	18 (10-34) (n = 43)	10 (6-25) (n = 22)	.12	16 (8-24)	17 (10-25)	10 (5-24)	.24
Disposition at hospital discharge								
Home	31 (47.0)	28 (65.1)	3 (13.0)		14 (43.8)	13 (65.0)	1 (8.3)	<.001
Died	17 (25.8)	0	17 (73.9)		10 (31.3)	0	10 (83.3)	
Inpatient rehabilitation	14 (21.2)	13 (30.2)	1 (4.4)		7 (21.9)	6 (30.0)	1 (8.3)	
Transfer to other hospital	1 (1.5)	1 (2.3)	0		0	0	0	
Long term care facility	3 (4.6)	1 (2.3)	2 (8.7)		1 (3.1)	1 (5.0)	0 (0.0)	
Time from cardiac arrest event to death, median (IQR), d^c^	10 (4-23) (n = 19)	NA	10 (4-23) (n = 19)		9 (3-23)	NA	9 (3-23) (n = 11)	NA
Cause of death up to 1y^c^			N = 19				N = 11	
Brain death	6 (31.6)	NA	6 (31.6)	NA	5 (45.5)	NA	5 (45.5)	NA
Multiple organ failure	6 (31.6)	NA	6 (31.6)		4 (36.4)	NA	4 (36.4)	
Neurologic injury	5 (26.3)	NA	5 (26.3)		2 (18.2)	NA	2 (18.2)	
Cardiovascular	2 (10.5)	NA	2 (10.5)		0 (0.0)	NA	0 (0.0)	
Pediatric Index of Mortality score, median (IQR)	0.15 (0.08-0.26)	0.14 (0.05-0.21)	0.20 (0.15-0.88)	<.001	0.17 (0.14-0.40)	0.15 (0.13-0.24)	0.42 (0.15-0.91)	.05
First Glasgow Coma Scale score in the intensive care unit, median (IQR)^c^	3 (3-7) (n = 46)	3 (3-8) (n = 25)	3 (3-6) (n = 21)	.18	3 (3-6) (n = 19)	3 (3-6) (n = 20)	3 (3-6) (n = 12)	.70
Extracorporeal membrane oxygenation	14 (21.2)	13 (30.2)	1 (4.4)	.02	4 (12.5)	4 (20.0)	0	.27
Extracorporeal cardiopulmonary resuscitation^c^	12 (85.7) (n = 14)	11 (84.6) (n = 13)	1 (100.0) (n = 1)	>.99	3 (75.0) (n = 4)	3 (75.0) (n = 4)	0	
Target temperature management, prevention of fever	20 (30.3)	13 (30.2)	7 (30.4)	>.99	7 (21.9)	6 (30.0)	1 (8.3)	.21
Target temperature management, therapeutic hypothermia	12 (18.2)	9 (20.9)	3 (13.0)	.52	8 (25.0)	5 (25.0)	3 (25.0)	>.99
Duration at target temperature, median (IQR), h^c^	60.0 (32.5-89.0) (n = 12)	58.0 (41.0-82.0) (n = 9)	62.0 (19.0-130.0) (n = 3)	>.99	44.5 (21.5-79.0) (n = 8)	41.0 (24.0-48.0) (n = 5)	62.0 (19.0-130.0) (n = 3)	.55

Abbreviations: MRI, magnetic resonance imaging; MRS, magnetic resonance spectroscopy; NA, not applicable.

^a^ A favorable outcome was defined as a Vineland Adaptive Behavioral Scale-3 (VABS-3) score ≥ 70 and an unfavorable outcome was defined as a VABS score < 70 or patient death.

^b^ *P* values are based on χ^2^ test for categorical variables and a Kruskal-Wallis test was used for medians.

^c^ Numbers in these rows differ from column totals because data were not available for some patients.

### Brain MRI Results by Outcome Group

MRI was performed at a median (IQR) of 5.0 (2.0-9.0) days after cardiac arrest and was performed earlier in children with an unfavorable outcome than in children with a favorable outcome (median [IQR], 2.0 [2.0-4.0] days vs 5.0 [3.0-12.0] days). Overall, brain lesions of any severity on T2-weighted imaging were observed most frequently in the cortex, specifically in the parietal lobe (22 children [33.3%]) and frontal lobe (21 children [31.8%]). Compared with children who had a favorable outcome, children with an unfavorable outcome more frequently had lesions in the thalamus (15 children [65.2%] vs 5 children [11.6%]),basal ganglia lenticular nucleus (13 children [56.5%] vs 5 children [11.6%]), and basal ganglia caudate nucleus (11 children [47.8%] vs 3 children [7.0%]) (eTable 3 in Supplement 1). Nearly one-third (19 children [28.8%]) of children had evidence of cerebral edema present on brain MRI, including in 9 children (20.9%) with a favorable outcome and in 10 children (43.5%) with an unfavorable outcome. Overall, brain lesions of any severity on DWI were seen most frequently in the frontal lobe (27 children [40.9%]) and parietal lobe (24 children [36.4%]). Notably, DWI lesions were significantly more frequent in all regions evaluated in children with an unfavorable vs favorable outcome except for the posterior limb of the internal capsule (eTable 3 in Supplement 1).

Analyzing these same regions by the severity of lesions, temporal lobe gray matter, parietal lobe white matter, basal ganglia, thalamus, and centrum semiovale scores were higher (ie, indicating worse outcomes) for children with an unfavorable outcome than for children with a favorable outcome (Table 3). DWI lesions in all 4 lobes, in both gray and white matter, basal ganglia, thalamus, cerebellar, brain stem white matter, and centrum semiovale were associated with an unfavorable outcome.

**Table 3.** Regional and Summated Brain MRI Scores Overall and by 1-Year Outcome^a^

MRI score	T2-weighted MRI score, median (IQR)				DWI score, median (IQR)			
	All (N = 66)	Favorable outcome (n = 43)	Unfavorable outcome (n = 23)	*P* value	All (N = 66)	Favorable outcome (n = 43)	Unfavorable outcome (n = 23)	*P* value
MRI regional score^b^								
Frontal lobe gray matter	0 (0-0)	0 (0-0)	0 (0-1)	.39	0 (0-0)	0 (0-0)	0 (0-2)	<.001
Frontal lobe white matter	0 (0-0)	0 (0-0)	0 (0-1)	.42	0 (0-1)	0 (0-0)	1 (0-3)	<.001
Temporal lobe gray matter	0 (0-0)	0 (0-0)	0 (0-1)	.04	0 (0-0)	0 (0-0)	0 (0-2)	.002
Temporal lobe white matter	0 (0-0)	0 (0-0)	0 (0-0)	.23	0 (0-0)	0 (0-0)	0 (0-3)	.001
Parietal lobe gray matter	0 (0-1)	0 (0-0)	0 (0-1)	.36	0 (0-1)	0 (0-0)	1 (0-2)	<.001
Parietal lobe white matter	0 (0-1)	0 (0-0)	0 (0-1)	.04	0 (0-1)	0 (0-0)	1 (0-3)	<.001
Occipital lobe gray matter	0 (0-0)	0 (0-0)	0 (0-1)	.29	0 (0-0)	0 (0-0)	0 (0-3)	<.001
Occipital lobe white matter	0 (0-0)	0 (0-0)	0 (0-1)	.17	0 (0-0)	0 (0-0)	1 (0-3)	<.001
Basal ganglia: lenticular	0 (0-1)	0 (0-0)	1 (0-3)	<.001	0 (0-0)	0 (0-0)	0 (0-3)	<.001
Basal ganglia: caudate	0 (0-0)	0 (0-0)	0 (0-3)	<.001	0 (0-0)	0 (0-0)	0 (0-3)	<.001
Thalamus gray matter	0 (0-2)	0 (0-0)	2 (0-3)	<.001	0 (0-1)	0 (0-0)	1 (0-3)	<.001
Cerebellum gray matter	0 (0-0)	0 (0-0)	0 (0-0)	.37	0 (0-0)	0 (0-0)	0 (0-0)	.21
Cerebellum white matter	0 (0-0)	0 (0-0)	0 (0-0)	.39	0 (0-0)	0 (0-0)	0 (0-0)	.03
Brain stem gray matter	0 (0-0)	0 (0-0)	0 (0-0)	.12	0 (0-0)	0 (0-0)	0 (0-0)	.06
Brain stem white matter	0 (0-0)	0 (0-0)	0 (0-0)	.43	0 (0-0)	0 (0-0)	0 (0-1)	.02
Posterior limb of the internal capsule white matter	0 (0-0)	0 (0-0)	0 (0-0)	.55	0 (0-0)	0 (0-0)	0 (0-2)	.11
Centrum semiovale white matter	0 (0-0)	0 (0-0)	0 (0-1)	.003	0 (0-1)	0 (0-0)	1 (0-3)	<.001
MRI summary scores								
Cortex score^c^	0 (0-4)	0 (0-3)	1 (0-6)	.10	0 (0-5)	0 (0-2)	8 (0-18)	<.001
Deep gray matter score^d^	0 (0-2)	0 (0-0)	6 (0-9)	<.001	0 (0-2)	0 (0-0)	2 (0-9)	<.001
T2-weighted imaging total score^e^	2 (0-8)	0 (0-4)	6 (1.0-14)	.002	NA	NA	NA	NA
DWI total score^e^	NA	NA	NA	NA	1.5 (0-13)	0 (0-3)	15 (3-23)	<.001
MRI Injury Score^f^	6 (0-22)	1 (0-8)	22 (7-32)	<.001	NA	NA	NA	NA

Abbreviations: DWI, diffusion-weighted imaging; MRI, magnetic resonance imaging; NA, not applicable.

^a^ A favorable outcome was defined as a Vineland Adaptive Behavioral Scale-3 (VABS-3) score 70 or higher, and an unfavorable outcome was defined as a VABS score less than 70 or patient death. The Kruskal-Wallis test was used to test differences between outcome groups.

^b^ MRI regions were scored by 2 pediatric neuroradiologists using this severity key: 1 = mild (<25% of region affected); 2 = moderate (25%-50% of region affected); and 3 = severe (>50% of region affected).

^c^ The cortex score is the sum of gray and white matter regional scores from the 4 cortical lobes (frontal, temporal, parietal, and occipital).

^d^ The deep gray matter score is the sum of basal ganglia (lenticular and caudate) and thalamus regional scores.

^e^ The total score is the sum of all regions for either T2-weighted imaging or DWI.

^f^ The MRI Injury Score is the sum of all regions for both T2-weighted imaging and DWI sequences.

All summary MRI Injury Scores except the T2-weighted imaging cortex score were significantly higher in children with an unfavorable outcome compared to children with a favorable outcome (Table 3). Overall, the MRI Injury Score, a sum of T2-weighted imaging and DWI findings together, was markedly increased in children with an unfavorable outcome than in children with a favorable outcome (median [IQR] score, 22 [7-32] vs 1 [0-8]).

### Brain MRS Results by Outcome Group

MRS was performed at a median (IQR) of 5.0 (3.5-9.5) days after cardiac arrest and was performed earlier in children with an unfavorable outcome than in children with a favorable outcome (median [IQR], 4.0 [2.0-5.5] days vs 7.5 [5.0-13.0] days) (Table 2). Increased lactate and decreased NAA were associated with an unfavorable outcome in all 4 ROIs (Figure and eTable 4 in Supplement 1). Similarly, the lactate-to-NAA ratio was significantly higher in children with an unfavorable outcome in all 4 ROIs.

**Figure.**
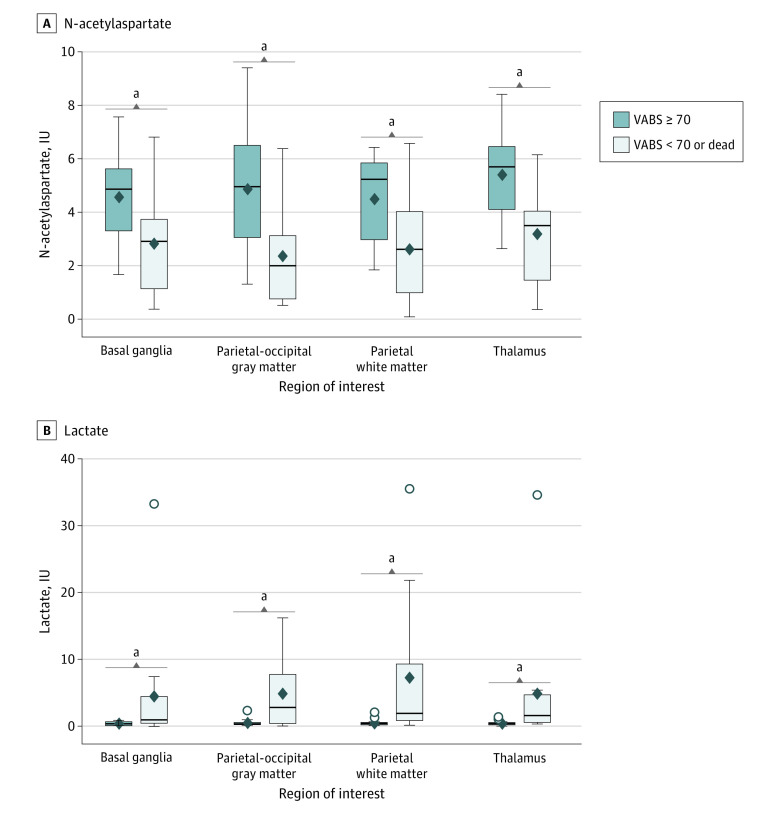
Magnetic Resonance Spectroscopy (MRS) of N-Acetylaspartate and Lactate Concentrations by 1-Year Outcome in 4 Regions of Interest Panel A shows MRS N-acetylaspartate concentrations, and panel B shows MRS lactate concentrations in 4 different regions of the brain at 1 year. Diamonds denote means, lines within boxes denote medians, tops and bottoms of boxes denote IQRs, error bars denote 95% CIs, and circles denote outliers. IU indicates institutional units; VABS, Vineland Adaptive Behavioral Scale. ^a^Denotes a significant difference (*P* < .05) between unfavorable vs favorable outcomes at 1 year.

### Association of MRI and MRS Features With 1-Year Outcomes

MRI Injury Score (odds ratio [OR], 1.11; 95% CI, 1.04-1.17) and witnessed event status (OR, 0.08; 95% CI, 0.02-0.30) were associated with 1-year outcomes in univariate analysis (eTable 5 in Supplement 1). After adjustment for patient age and sex, MRI Injury Score (OR, 1.12; 95% CI, 1.04-1.20) and witnessed event status (OR, 0.07; 95% CI, 0.01-0.34) remained associated with 1-year outcomes. Multivariable AUROC for this model was 0.886 (95% CI, 0.786-0.985), and was more accurate than the AUROC for clinical variables alone (0.755; 95% CI, 0.623-0888; *P* = .03). Next, deep gray matter injury scores for both T2-weighted imaging (OR, 1.70; 95% CI, 1.25-2.31) and DWI (OR, 2.01; 95% CI, 1.32-3.07) sequences were associated with 1-year outcomes in multivariable analysis and had the highest AUROC values (AUROC for T2-weighted imaging; 0.888 [95% CI, 0.793-0.984]; AUROC for DWI, 0.900 [95% CI, 0.811-0.988]) among summated scores. Univariate logistic regression analysis of NAA and lactate in all 4 ROIs were all associated with 1-year outcomes, (eTable 6 in Supplement 1).

### Case Studies

In Supplement 1, eFigure 2 and eTable 7 highlight 3 patient cases. These cases are representative of more severe injury, moderately severe injury, and no injury at 1 year.

## Discussion

In this cohort study of children with cardiac arrest, brain MRI and MRS features from sequences obtained within the first 2 weeks after resuscitation were associated with 1-year outcomes. Our main findings were that (1) lesions observed in a majority of brain regions using DWI and T2-weighted MRI (to a lesser degree) were associated with 1-year outcomes; (2) higher MRI injury and DWI deep gray matter injury scores were associated with an unfavorable 1-year outcome in multivariable models; and (3) decreased NAA, increased lactate, and the lactate-to-NAA ratio were associated with an unfavorable 1-year outcome in all ROIs in univariate analyses.

Brain MRI is commonly used to evaluate hypoxic-ischemic injury following pediatric cardiac arrest, but evidence regarding best practices in terms of sequences and timing postarrest is lacking.^1,4,24^ Lesions on DWI sequences may become apparent as early as a few hours after arrest, peak at 3 to 7 days after arrest, and then may recede.^25^ MRS metabolites may offer better stability over time.^26^ In practice, however, children are not always clinically stable (eg, vasoactive requirement) or safe (eg, extracorporeal membrane oxygenation) to undergo brain MRI or MRS in the first few days after arrest. Conversely, for a stable patient, a clinician may decide the benefit is not worth the risks and costs (eg, intrahospital transport, need for multiple intensive care unit clinicians’ time off the unit, and/or additional medications necessary for procedural facilitation).^27,28^ Thus, our study analyzed the earliest clinically performed test occurring within the first 2 weeks after arrest. Prior studies^6,7,29,30,31^ have shown that cytotoxic injury occurs most frequently in regions with increased vulnerability to hypoxia-ischemia, especially the occipital and parietal lobes and basal ganglia. Our MRI findings, conducted, to our knowledge, for the first time in a multicenter study, confirm these injury patterns. Furthermore, our study provides a more detailed accounting of regional gray and white matter injury location and severity, adapting a clinically relevant scoring system from the adult cardiac arrest literature.^16^

Prior single-center studies^6,31,32^ examining brain MRI and MRS as prognostic tools varied in terms of patient population (eg, mix of children with cardiac arrest along with other neurologic conditions or only drowning patients), imaging details (eg, timing, magnet strength, and scoring), and outcome assessment (eg, outcome measures and outcome timing). Those foundational studies identified specific brain regions (watershed regions,^31^ generalized and occipital cortex edema,^32^ and basal ganglia^6^) associated with outcomes using T2-weighted imaging. DWI is another sequence that is performed regularly as the standard of care in this population and may be a more robust factor associated with outcomes. In an earlier study^6^ at our center, restricted diffusion in cortical gray and white matter and basal ganglia was also associated with outcomes after pediatric cardiac arrest. Another study^33^ that evaluated DWI in children with cardiac arrest found that lesions in the basal ganglia and cortex were associated with an unfavorable outcome, and that measured global apparent diffusion coefficient values of less than 31% of uninjured white matter also were associated with a poor outcome. Finally, in another recent single-center study^7^, investigators adapted a validated stroke imaging tool to quantitate injury with DWI using clinically indicated MRI performed within the first 2 weeks of the event. They found an AUROC of 0.96 for DWI and 0.92 for T2-weighted imaging to differentiate favorable and unfavorable outcomes in children with out-of-hospital cardiac arrest. Our study affirmed the importance of imaging findings across a multicenter cohort of children who had a cardiac arrest and further suggests that MRS may provide additional key information.

There are no MRI scoring tools validated for outcomes after pediatric cardiac arrest. We adapted an adult cardiac arrest MRI scoring tool for pediatric use given its use of both T2-weighted and DWI sequences, elements specific to cardiac arrest, incorporation of lesion severity of both white and gray matter, and validity in adults.^16^ In this study, we were able to show an association of MRI Injury Score with outcomes, which can be used to support shared decision-making. In POCCA,^12^ the MRI Injury and DWI Deep Gray Matter Scores were associated with our study’s primary outcome at 1 year after adjustment for a limited number of clinical variables due to sampling size, with excellent accuracy. External validation of these scores in a new cohort is essential to confirm clinical capacity.

Finally, MRS has potential to improve diagnostic accuracy for children with cardiac arrest. Decreased NAA-to-creatine ratios and increased lactate in the occipital gray and parietal white cortical matter on brain MRS were associated with worse outcome in studies of children with cardiac arrest and other acute neurologic conditions in seminal studies in the 1990s.^17^ Lesions on MRI were correlated with altered metabolites on MRS in children who had cardiac arrest due to drowning.^32^ Our pilot work confirmed these findings in the basal ganglia and thalamus.^8^ Our results in POCCA^12^ strengthen the potential utility for MRS as an objective prognostic biomarker in pediatric cardiac arrest. Still, more work is needed to demonstrate that MRS improves the diagnostic accuracy of clinical variables and conventional MRI. A meta-analysis^34,35^ in neonates with birth asphyxia that compared various imaging modalities showed the lactate-to-NAA ratio had high sensitivity (82%) and specificity (95%) and was associated with neurodevelopmental outcomes.

### Strengths and Limitations

Strengths of our study include the multicenter design, individual site training, and harmonizing of MRI and MRS protocols for each center’s platform. Clinically, brain MRI is commonly used to evaluate postcardiac arrest as described in an international survey^24^. Nearly one-half of the respondents reported performing a brain MRI for any surviving child and one-third of respondents reported performing a brain MRI for children with newly diagnosed impairment. Almost all responding center personnel reported performing conventional and DWI primary MRI sequences with fewer than one-third performing MRS. Once validated, our data may help inform future evidence-based guidelines for using and interpreting of MRI for prognostication after pediatric cardiac arrest. Future directions also include determining whether brain MRI and MRS improves diagnostic accuracy when in combination with key clinical variables, blood-based biomarkers, and other clinically relevant testing (eg, electroencephalography).

This study has some limitations. Patient and arrest variables in the overall parent study population^12^ for patients who had or did not undergo brain MRI is in eTable 1 in Supplement 1. Notably, patients with longer arrest duration, who were more deeply comatose after arrest, and who had more hospital disposition to rehabilitation had MRI performed. Patients with an unfavorable outcome had MRI or MRS assessed earlier than those with a favorable outcome, potentially biasing MRI and MRS findings. Our analysis may thus not represent all lesions given their natural evolution over time.^27^ Indeed, given that imaging occurred earlier in patients with unfavorable outcomes suggests that in some cases, the clinical decision to image is not based solely on patient stability. Intensive care unit clinicians determined whether a brain MRI or MRS was clinically indicated. We did not collect information on why a brain MRI or MRS was not performed. The use of clinically indicated brain imaging may have introduced bias. Clinicians were aware of imaging findings, potentially introducing bias in clinical care that could be associated with outcomes. Sample size remains a limitation, specifically for high-quality MRS data. In this study, MRS ROIs were selected according to the availability of substantial control data regarding normal age-dependent metabolic changes. It is conceivable that the metabolic evaluation of brain injury can be improved by selecting a different set of ROIs placed in brain regions that are more associated with long-term outcomes. Outcome analysis was not adjusted for prearrest baseline function, but entry criteria excluded children with severe coma at baseline. The POCCA protocol^12^ did not record who died due to withdrawal from life-sustaining therapy.

## Conclusions

In this cohort study of pediatric patients with cardiac arrest who underwent MRI and MRS within 2 weeks, higher MRI Injury Score, increased lactate, and decreased NAA in regions of interest were associated with an unfavorable outcome at 1 year. External validation is needed to confirm these findings for clinical implementation.

## Notes

Supplement 1. **eFigure 1.** Study Flowchart **eTable 1.** Demographics, Cardiac Arrest and Resuscitation, Treatment, and Outcome Variables Compared Between Children With and Without Brain MRI Performed From the Overall Primary Study Cohort **eAppendix 1.** Magnetic Resonance Imaging and Spectroscopy Procedures and Protocols for Data Acquisition, Image Extraction, and Anonymization **eAppendix 2.** MRI Scoring **eAppendix 3.** 4 Regions of Interest **eTable 2.** Simple and Weighted Kappa Scores by Brain Region **eTable 3.** Frequency of Lesions of Any Severity on Brain T2-Weighted Magnetic Resonance Imaging and Diffusion Weighted-Imaging in the Overall Group and by Favorable and Unfavorable Outcome at 1 Year **eTable 4.** Brain Magnetic Resonance Spectroscopy Concentrations of N-Acetylaspartate and Lactate Expressed Individually and as a Ratio of Lactate: N-Acetylaspartate in 4 Regions of Interest in the Overall Cohort and by Favorable and Unfavorable Outcome at 1 Year **eTable 5.** Univariate and Stepwise, Multivariable Logistic Regressions for the Association Between Magnetic Resonance Imaging (MRI) and 1 Year Outcome **eTable 6.** Univariate Logistic Regression and Area Under the Receiver Operator Curves (AUROC) **eFigure 2.** Representative Patient Brain Magnetic Resonance Imaging and Spectroscopy of Children With Cardiac Arrest With More Severe Impairment (A), Moderately Severe Impairment (B), and No Impairment (C) at 1 Year **eTable 7.** Imaging Details for the Patients A, B, and C in eFigure 2

Supplement 2.
Nonauthor Collaborators

Supplement 3.
Data Sharing Statement
