# Supplementary material for: Assessment of Brain Magnetic Resonance and Spectroscopy Imaging Findings and Outcomes After Pediatric Cardiac Arrest
Source: JAMA Netw Open. 2023 Jun 30;6(6):e2320713. doi: 10.1001/jamanetworkopen.2023.20713 (PMC10314315; doi:10.1001/jamanetworkopen.2023.20713)
Supplement: Supplement 1. — eFigure 1. Study Flowchart eTable 1. Demographics, Cardiac Arrest and Resuscitation, Treatment, and Outcome Variables Compared Between Children With and Without Brain MRI Performed From the Overall Primary Study Cohort eAppendix 1. Magnetic Resonance Imaging and Spectroscopy Procedures and Protocols for Data Acquisition, Image Extraction, and Anonymization eAppendix 2. MRI Scoring eAppendix 3. 4 Regions of Interest eTable 2. Simple and Weighted Kappa Scores by Brain Region eTable 3. Frequency of Lesions of Any Severity on Brain T2-Weighted Magnetic Resonance Imaging and Diffusion Weighted-Imaging in the Overall Group and by Favorable and Unfavorable Outcome at 1 Year eTable 4. Brain Magnetic Resonance Spectroscopy Concentrations of N-Acetylaspartate and Lactate Expressed Individually and as a Ratio of Lactate: N-Acetylaspartate in 4 Regions of Interest in the Overall Cohort and by Favorable and Unfavorable Outcome at 1 Year eTable 5. Univariate and Stepwise, Multivariable Logistic Regressions for the Association Between Magnetic Resonance Imaging (MRI) and 1 Year Outcome eTable 6. Univariate Logistic Regression and Area Under the Receiver Operator Curves (AUROC) eFigure 2. Representative Patient Brain Magnetic Resonance Imaging and Spectroscopy of Children With Cardiac Arrest With More Severe Impairment (A), Moderately Severe Impairment (B), and No Impairment (C) at 1 Year eTable 7. Imaging Details for the Patients A, B, and C in eFigure 2 [file jamanetwopen-e2320713-s001.pdf]

Supplemental Online Content

Fink EL, Kochanek PM, Beers SR, et al; POCCA Investigators. Assessment of brain magnetic resonance and spectroscopy imaging findings and outcomes after pediatric cardiac arrest. *JAMA Netw Open*. 2023;6(6):e2320713. doi:10.1001/jamanetworkopen.2023.20713

eFigure 1. Study Flowchart
eTable 1. Demographics, Cardiac Arrest and Resuscitation, Treatment, and Outcome Variables Compared Between Children With and Without Brain MRI Performed From the Overall Primary Study Cohort
eAppendix 1. Magnetic Resonance Imaging and Spectroscopy Procedures and Protocols for Data Acquisition, Image Extraction, and Anonymization
eAppendix 2. MRI Scoring
eAppendix 3. 4 Regions of Interest
eTable 2. Simple and Weighted Kappa Scores by Brain Region
eTable 3. Frequency of Lesions of Any Severity on Brain T2-Weighted Magnetic Resonance Imaging and Diffusion Weighted-Imaging in the Overall Group and by Favorable and Unfavorable Outcome at 1 Year
eTable 4. Brain Magnetic Resonance Spectroscopy Concentrations of N-Acetylaspartate and Lactate Expressed Individually and as a Ratio of Lactate: N-Acetylaspartate in 4 Regions of Interest in the Overall Cohort and by Favorable and Unfavorable Outcome at 1 Year
eTable 5. Univariate and Stepwise, Multivariable Logistic Regressions for the Association Between Magnetic Resonance Imaging (MRI) and 1 Year Outcome
eTable 6. Univariate Logistic Regression and Area Under the Receiver Operator Curves (AUROC)
eFigure 2. Representative Patient Brain Magnetic Resonance Imaging and Spectroscopy of Children With Cardiac Arrest With More Severe Impairment (A), Moderately Severe Impairment (B), and No Impairment (C) at 1 Year
eTable 7. Imaging Details for the Patients A, B, and C in eFigure 2

This supplemental material has been provided by the authors to give readers additional information about their work.

© 2023 Fink EL et al. *JAMA Network Open.*

eFigure 1. Study Flowchart.

MRI, magnetic resonance imaging; MRS, magnetic resonance spectroscopy; PCPC, pediatric cerebral performance category; ICU, intensive care unit; LASER, localization by adiabatic selective refocusing

* may have more than one reason

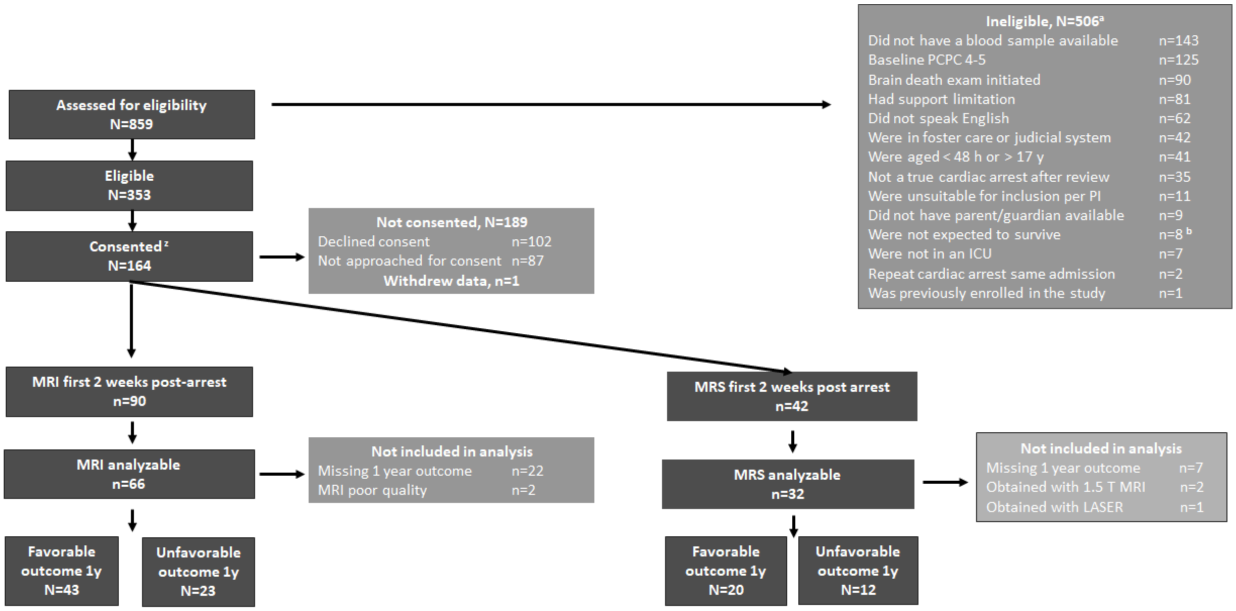

© 2023 Fink EL et al. *JAMA Network Open.*

eTable 1. Demographics, Cardiac Arrest and Resuscitation, Treatment, and Outcome Variables Compared Between Children With and Without Brain MRI Performed From the Overall Primary Study Cohort.

**Characteristics**	**Overall**<br><br>**N=163**	**Without Brain MRI**<br><br>**N=75**	**With Brain MRI**<br><br>**N=88**	**p-value**
**Age, years**	1.0 (0.0, 9.0)	2.0 (0.0, 11.0)	1.0 (0.0, 4.5)	0.16
**Sex**				0.81
Female	69 (42.3)	31 (41.3)	38 (43.2)	
Male	94 (57.7)	44 (58.7)	50 (56.8)	
**Race**				0.43
African American/Black	36 (22.1)	21 (28.0)	15 (17.0)	
Asian	5 (3.1)	2 (2.7)	3 (3.4)	
White	104 (63.8)	46 (61.3)	58 (65.9)	
Unknown	18 (12.0)	6 (8.0)	12 (13.6)	
**Hispanic Ethnicity**	14 (9.2), n=152	4 (5.9), n=68	10 (11.9), n=84	0.20
**Pre-existing conditions**	103 (66.0), n=156	53 (73.6), n=72	50 (59.5), n=84	0.06
**Primary etiology**	N=148	N=72	N=76	0.44
Asphyxia	108 (73.0)	51 (70.8)	57 (75.0)	
Cardiac	40 (27.0)	21 (29.2)	19 (25.0)	
**Event location**				0.16
Out-of-hospital	90 (55.2)	37 (49.3)	53 (60.2)	
In-hospital	73 (44.8)	38 (50.7)	35 (39.8)	

© 2023 Fink EL et al. *JAMA Network Open.*

**Duration of cardiopulmonary resuscitation, min**	7.0 (2.5, 20.0), n=132	3.5 (2.0, 10.0), n=60	11.0 (5.0, 23.5), n=72	<0.001
**Total number of epinephrine doses**	1.0 (0.0, 3.0), n=137	1.0 (0.0, 3.0), n=64	2.0 (1.0, 3.0), n=73	0.09
**Defibrillated**	25 (18.0), n=139	12 (19.4), n=62	13 (16.9), n=77	0.71
**First monitored rhythm**	n=124	n=55	n=69	0.57
Sinus bradycardia	44 (35.5)	17 (30.9)	27 (39.1)	
Pulseless electrical activity	29 (23.4)	16 (29.1)	13 (18.8)	
Asystole	20 (16.1)	7 (12.7)	13 (18.8)	
Ventricular tachycardia or fibrillation	18 (14.5)	9 (16.4)	9 (13.0)	
Other (normal sinus, sinus tachycardia)	13 (10.5)	6 (10.9)	7 (10.1)	
**Witnessed status**	121 (74.2)	58 (77.3)	63 (71.6)	0.40
**Bystander resuscitation**				0.57
Healthcare personnel	108 (66.3)	48 (64.0)	60 (68.2)	
Non-healthcare personnel	55 (33.7)	27 (36.0)	28 (31.8)	
**Hospital length of stay, days**	15.0 (6.0, 33.0), n=162	8.0 (4.0, 30.0), n=75	18.0 (12.0, 39.0), n=87	0.001
**Intensive care unit length of stay, days**	11.0 (5.0, 21.0), n=157	7.0 (3.5, 15.5), n=72	13.0 (8.0, 25.0), n=85	0.001
**Disposition at hospital discharge**	N=162	N=75	N=87	0.01

© 2023 Fink EL et al. *JAMA Network Open.*

Home	88 (54.3)	41 (54.7)	47 (54.0)	
Died	41 (25.3)	24 (32.0)	17 (19.5)	
Inpatient rehabilitation	23 (14.2)	4 (5.3)	19 (21.8)	
Transfer to other hospital	5 (3.1)	4 (5.3)	1 (1.1)	
Long term care facility	5 (3.1)	2 (2.7)	3 (3.4)	
**Days from cardiac arrest event to death**	10.0 (3.0, 25.0), n=43	8.5 (3.0, 25.5), n=24	10.0 (4.0, 23.0), n=19	0.56
**Cause of death up to 1y**	N=43	N=24	N=19	0.64
Brain death	11 (25.6)	5 (20.8)	6 (31.6)	
Multiple Organ Failure	13 (30.2)	7 (29.2)	6 (31.6)	
Neurologic injury	11 (25.6)	6 (25.0)	5 (26.3)	
Cardiovascular	8 (18.6)	6 (25.0)	2 (10.5)	
**Pediatric Index of Mortality score**	16.4 (9.8, 29.3)	17.8 (6.3, 35.2)	15.4 (11.0, 25.7)	0.27
**First Glasgow Coma Scale score in the ICU**	6.0 (3.0, 11.0), n=123	8.0 (3.0, 11.0), n=61	3.0 (3.0, 7.0), n=62	<0.001
**Extracorporeal membrane oxygenation**	32 (19.6)	14 (18.7)	18 (20.5)	0.78
Extracorporeal CPR	22 (68.8), n=32	10 (71.4), n=14	12 (66.7), n=18	0.77
**Target temperature management, prevention of fever**	52 (31.9)	20 (26.7)	32 (36.4)	0.19

© 2023 Fink EL et al. *JAMA Network Open.*

**Target temperature management, therapeutic hypothermia**	16 (9.8)	4 (5.3)	12 (13.6)	0.08
Duration at target temperature, h	58.5 (32.0, 82.0), n=16	49.5 (24.0, 70.5), n=4	60.0 (32.5, 89.0), n=12	0.43

MRI, magnetic resonance imaging; ICU, intensive care unit

© 2023 Fink EL et al. *JAMA Network Open.*

eAppendix1. Magnetic Resonance Imaging and Spectroscopy Procedures and Protocols for Data Acquisition, Image Extraction, and Anonymization

A. Conventional and Diffusion Weighted MRI

1. Sagittal T1-SPGR or vendor equivalent of this sequence with voxel size 1.5 mm ^{3}; which can be reformatted into the axial plane. Optional T1 sequences include axial imaging plane oriented parallel to the AC*PC line and sagittal T1 @ 1.5T [Field-of-view (FOV) 200-240 mm; 4 mm slice thickness; 0.4 mm gap; TR/TE 500/12; Voxel 1.5 mm^3; and NSA 2-3. T1 FLAIR images should be acquired instead of T1 spin echo @ 3T
2. T2 axial imaging plane oriented parallel to the AC*PC line (FOV 200; TSE ~21; TR/TE 3500-4500/100-115; 4mm slice thickness, .4 gap; voxel 1.5mm^3; NSA 2-3.
3. The parameters for DWI include: FOV 210; TR shortest; TE 74-80; B= 1000; 3 directions; NSA 3; slice thickness 4-5 mm; Voxel 2.5-5 mm^3.

**Sagittal T1 SPGR/MPRAGE**	
MR Scanner	Philips, Siemens, General Electric
Field strength	1.5T, 3T
Coils	Multi-channel head coil OR single channel neonatal head coil.
Sequence:	T1 3D TFE (Philips); SPGR (GE); MP-RAGE (SIEMENS)
Imaging plane	Sagittal
Repetition time (TR)/echo time (TE):	Philips: shortest (9.9 msec)/ 4.6; GE/Siemens: 36 ms/min
Acquisition matrix	200x 137
FOV	200 x 200
Slice thickness (z-dimension):	1 mm
Voxel	1 x1 x mm isotropic
Flip angle	8
Multi-shot TFE factor	109; shot interval shortest
Acquisitions	2
Fat suppression	No
Acceleration factor	SENSE 1.3; ASSET 1.0
Acquisition time:	4:39 sec
TI delay	594

© 2023 Fink EL et al. *JAMA Network Open.*

**T2 Fast spin echo**	
MR Scanner	Philips, Siemens, General Electric
Field strength	1.5 and 3.0T
Coils	Quadrature head, multi-channel head coil OR single channel neonatal head coil
Sequence:	T2 TSE, FSE
Imaging plane	Axial
Repetition time (TR)/echo time (TE):	3500-4500/90-120
FOV:	~200 x 200
Slice thickness (z-dimension):	3-4 mm; 10% interslice gap
Voxel	0.7-1 x 0.7-1 x 4mm
Multi-shot TSE factor	Philips 19
Acquisitions	2-4
Fat suppression	No
Acceleration factor	SENSE 1.3; ASSET 1.0
Acquisition time:	4:22 sec
Accleration factor	0-1

**DWI**	
MR Scanner	Philips, Siemens, General Electric
Field strength	1.5 and 3.0T
Coils	Quadrature, multi-channel head coil OR single channel neonatal head coil
Sequence:	DWI
Imaging plane	Axial
Repetition time (TR)/echo time (TE):	TR shortest; TE 74-80
Acquisition	2
FOV:	200 x 200
Slice thickness (z-dimension):	4-5 mm
Voxel	1 x 1 x mm isotropic
B value	1000
Acquisitions	2
Fat suppression	No
Acceleration factor	SENSE 1.3; ASSET 1.0
Acquisition time	56 sec.

© 2023 Fink EL et al. *JAMA Network Open.*

B. Magnetic Resonance Spectroscopy (MRS)

1. Single voxel (SV) MRS will be utilized in this study.
2. Both metabolite and water spectra were acquired with a short echo time TE = 35ms.
3. There were 4 regions of interest (ROI) in this study:
    a. Basal ganglia
    b. Thalamus
    c. Parietal white matter
    d. Occipitoparietal cortex
4. Sequence and sequence parameters

**MR Scanner**	**General Electric, Philips, Siemens**
Field strength	1.5T and 3.0T
Coils	Use head coil. Multiple receivers/ phased array coils are okay to use.
Nuclei	^1H
Sequence:	PRESS-Point resolved spectroscopy sequence
Repetition time (TR):	1.5s (1.5T) 2.0s (3.0T)
Echo time (TE):	35 ms (metabolite and water spectrum)
FOV:	n.a. (default)
Phase encoding:	1 × 1 (= single voxel mode)
Spectral bandwidth	2000 Hz
Number of spectral points	1024
*Slice thickness (z-dimension):	10-20mm
*In-plane (xy) dimensions	10-20 × 10-20 mm^2
*Volume of region of interest (ROI)	3-5cm^3 (< 3months), 5-8cm^3 (>3 years)
Averages for metabolite spectrum:	128
**Averages for water spectrum:	16
Water suppression (metabolite spectrum)	Water sat (Siemens), standard (GE), standard/excitation (Philips)
Water suppression (water spectrum)	"Off"
Shimming method	Brain (Siemens), auto (GE), pencil beam (Philips)
Outer volume suppression/ saturation bands	No.
Acquisition time:	5min/spectrum (incl. scanner adjustments and reference water scans)

*Volume = z-dim × x-dim × y-dim
**Only if separate scans to obtain water spectra are required

© 2023 Fink EL et al. *JAMA Network Open.*

eAppendix2. MRI Scoring.

Area		Region	Item #	T2	DWI
**Supratentorial**					
Gray matter	Cortex	Frontal	1	0 to 3	0 to 3
		Parietal	2	0 to 3	0 to 3
		Temporal	3	0 to 3	0 to 3
		Occipital	4	0 to 3	0 to 3
	Deep gray	Lenticular	5	0 to 3	0 to 3
		Caudate	6	0 to 3	0 to 3
		Thalamus	7	0 to 3	0 to 3
White matter	Cortex	Frontal	8	0 to 3	0 to 3
		Parietal	9	0 to 3	0 to 3
		Temporal	10	0 to 3	0 to 3
		Occipital	11	0 to 3	0 to 3
**Infratentorial**					
Gray matter		Brainstem	12	0 to 3	0 to 3
		Cerebellum	13	0 to 3	0 to 3
White matter		Brainstem	14	0 to 3	0 to 3
		Cerebellum	15	0 to 3	0 to 3
**White matter**					
		Posterior limb internal capsule (PLIC)	16	0 to 3	0 to 3
		Centrum semi-ovale	17	0 to 3	0 to 3

There are total 7 new variables for POCCA MRI summary scores:

**Variable**	**Calculation**	**Score Range**
T2 Cortex score	sum of item 1 to 4 and item 8 to 11 in T2 column	0 to 24
DWI Cortex score	sum of item 1 to 4 and item 8 to 11 in DWI column	0 to 24
T2 Deep gray score	Sum of item 5 to 7 in T2 column	0 to 12
DWI Deep gray score	Sum of item 5 to 7 in DWI column	0 to 12
T2 score	sum of item 1 to 17 in T2 column	0 to 51
DWI score	sum of item 1 to 17 in DWI column	0 to 51
Total MRI score	sum of items 1 to 17 in both T2 and DWI column	0 to 102

© 2023 Fink EL et al. *JAMA Network Open.*

eAppendix3. 4. Regions of Interest:
Typical size and location of basal ganglia, thalamus, occipitoparietal, and frontal white matter ROIs (**image, below**)

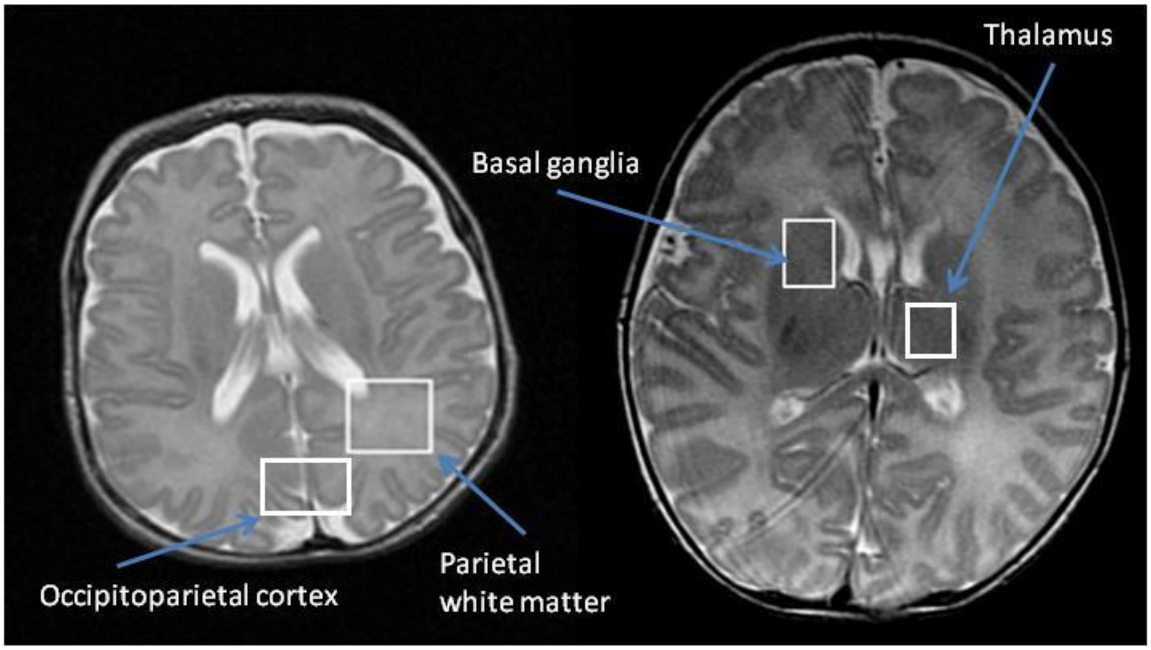

© 2023 Fink EL et al. *JAMA Network Open.*

eTable 2. Simple and Weighted Kappa scores by brain region. Two independent pediatric neuroradiologists blinded to each other's evaluations scored each brain MRI sequence and region below using the severity key: 1 = Mild, less than 25% of region affected; 2 = Moderate, 25-50% of region affected; 3 = Severe, > 50% of region affected.

	T2-weighted imaging		Diffusion-weighted imaging	
**Regional**	**Simple Kappa**	**Weighted Kappa**	**Simple Kappa**	**Weighted Kappa**
Frontal lobe: gray matter	0.35 (0.14, 0.57)	0.48 (0.24, 0.72)	0.51 (0.33, 0.69)	0.58 (0.39, 0.77)
Frontal lobe: white matter	0.24 (0.04, 0.44)	0.33 (0.12, 0.53)	0.35 (0.17, 0.53)	0.42 (0.21, 0.62)
Temporal lobe: gray matter	0.44 (0.20, 0.68)	0.52 (0.26, 0.78)	0.42 (0.19, 0.66)	0.52 (0.27, 0.77)
Temporal lobe: white matter	0.37 (0.13, 0.60)	0.36 (0.16, 0.55)	0.42 (0.22, 0.61)	0.38 (0.16, 0.59)
Parietal lobe: gray matter	0.52 (0.33, 0.72)	0.53 (0.30, 0.75)	0.54 (0.38, 0.70)	0.55 (0.37, 0.72)
Parietal lobe: white matter	0.27 (0.06, 0.49)	0.25 (0.04, 0.46)	0.38 (0.19, 0.57)	0.46 (0.25, 0.68)
Occipital lobe: gray matter	0.50 (0.30, 0.70)	0.64 (0.45, 0.82)	0.53 (0.37, 0.69)	0.69 (0.54, 0.83)
Occipital lobe: white matter	0.19 (0.00, 0.37)	0.27 (0.04, 0.49)	0.34 (0.15, 0.54)	0.49 (0.26, 0.71)
Basal ganglia: Lenticular	0.61 (0.44, 0.78)	0.64 (0.46, 0.83)	0.67 (0.49, 0.84)	0.71 (0.53, 0.90)
Basal ganglia: Caudate	0.66 (0.46, 0.86)	0.66 (0.45, 0.87)	0.61 (0.39, 0.84)	0.60 (0.35, 0.84)
Thalamus	0.49 (0.29, 0.69)	0.55 (0.32, 0.77)	0.68 (0.50, 0.86)	0.76 (0.59, 0.93)
Cerebellum: gray matter	0.37 (0.10, 0.63)	0.52 (0.21, 0.83)	0.27 (0.01, 0.54)	0.41 (0.08, 0.73)
Cerebellum: white matter	0.43 (0.10, 0.77)	0.51 0.11, 0.90)	0.28 (-0.05, 0.61)	0.31 (-0.09, 0.71)
Brain stem: gray matter	0.37 (0.10, 0.63)	0.45 (0.16, 0.74)	0.27 (0.01, 0.53)	0.42 (0.11, 0.74)
Brain stem: white matter	0.29 (0.01, 0.58)	0.34 (0.02, 0.66)	0.20 (-0.05, 0.45)	0.25 (-0.04, 0.53)
Posterior limb of the internal capsule	0.24 (-0.06, 0.55)	0.15 (-0.08, 0.39)	0.09 (-0.16, 0.33)	-0.00 (-0.13, 0.12)
Centrum semiovale	0.47 (0.20, 0.73)	0.34 (0.09, 0.59)	0.40 (0.16, 0.65)	0.42 (0.15, 0.70)
Cerebral edema	0.44 (0.22, 0.66)	0.55 (0.32, 0.77)	n/a	n/a
Brain herniation	0.55 (0.10, 1.00)	n/a	n/a	n/a

n/a, not applicable

© 2023 Fink EL et al. *JAMA Network Open.*

eTable 3. Frequency of Lesions of Any Severity on Brain T2-Weighted Magnetic Resonance Imaging and Diffusion Weighted-Imaging in the Overall Group and by Favorable and Unfavorable Outcome at 1 Year.

	**T2-weighted imaging**				**Diffusion-weighted imaging**			
Regional lesion	**All N=66**	**Favorable N=43**	**Unfavorable N=23**	**p-value**	**All N=66**	**Favorable N=43**	**Unfavorable N=23**	**p-value**
Frontal lobe[1]	21 (31.8)	11 (25.6)	10 (43.5)	0.17	27 (40.9)	11 (25.6)	16 (69.6)	<0.001
Temporal lobe[1]	14 (21.2)	5 (11.6)	9 (39.1)	0.01	17 (25.8)	5 (11.6)	12 (52.2)	<0.001
Parietal lobe[1]	22 (33.3)	11 (25.6)	11 (47.8)	0.10	24 (36.4)	8 (18.6)	16 (69.6)	<0.001
Occipital lobe[1]	17 (25.8)	8 (18.6)	9 (39.1)	0.08	19 (28.8)	5 (11.6)	14 (60.9)	<0.001
Basal ganglia: Lenticular	18 (27.3)	5 (11.6)	13 (56.5)	<0.001	14 (21.2)	3 (7.0)	11 (47.8)	<0.001
Basal ganglia: Caudate	14 (21.2)	3 (7.0)	11 (47.8)	<0.001	10 (15.2)	2 (4.7)	8 (34.8)	0.002
Thalamus	20 (30.3)	5 (11.6)	15 (65.2)	<0.001	17 (25.8)	3 (7.0)	14 (60.9)	<0.001
Cerebellum[1]	7 (10.6)	3 (7.0)	4 (17.4)	0.23	7 (10.6)	2 (4.7)	5 (21.7)	0.05
Brain stem[1]	10 (15.2)	5 (11.6)	5 (21.7)	0.30	13 (19.7)	5 (11.6)	8 (34.8)	0.05
Posterior limb of the internal capsule	7 (10.6)	4 (9.3)	3 (13.0)	0.69	11 (16.7)	5 (11.6)	6 (26.1)	0.17
Centrum semiovale	9 (13.6)	2 (4.7)	7 (30.4)	0.007	17 (25.8)	5 (11.6)	12 (52.2)	<0.001
Cerebral edema[2]	19 (28.8)	9 (20.9)	10 (43.5)	0.09				
Brain herniation[2]	4 (6.1)	1 (2.3)	3 (13.0)	0.12				

© 2023 Fink EL et al. *JAMA Network Open.*

Data are presented as n (%)

[1] Includes gray and white matter

[2] Assessed on T2 sequence only

© 2023 Fink EL et al. *JAMA Network Open.*

eTable 4. Brain Magnetic Resonance Spectroscopy Concentrations of N-Acetylaspartate and Lactate Expressed Individually and as a Ratio of Lactate: N-Acetylaspartate in 4 Regions of Interest in the Overall Cohort and by Favorable and Unfavorable Outcome at 1 Year.

**Region of interest, Metabolite, Median (IQR)**	All N=32	Favorable N=20	Unfavorable N=12	p-value
Basal ganglia, N-acetyl-aspartate	n=30	n=18	n=12	0.02
	3.7 (2.6, 5.4)	4.8 (3.3, 5.6)	2.9 (1.1, 3.7)	
Basal ganglia, Lactate	n=30	n=18	n=12	0.01
	0.5 (0.2, 0.9)	0.4 (0.1, 0.6)	1.0 (0.5, 4.5)	
Parietal-occipital gray matter, N-acetyl-aspartate	n=32	n=20	n=12	0.003
	3.9 (2.0, 5.7)	4.9 (3.1, 6.5)	2.0 (0.8, 3.1)	
Parietal-occipital gray matter, Lactate	n=32	n=20	n=12	0.008
	0.4 (0.3, 1.5)	0.3 (0.3, 0.6)	2.8 (0.4, 7.7)	
Parietal-occipital white matter, N-acetyl-aspartate	n=29	n=17	n=12	0.02
	3.6 (2.1, 5.5)	5.2 (2.9, 5.8)	2.6 (1.0, 4.0)	
Parietal-occipital white matter, Lactate	n=29	n=17	n=12	0.001
	0.5 (0.4, 1.5)	0.4 (0.2, 0.5)	1.9 (0.8, 9.3)	
Thalamus, N-acetyl-aspartate	n=27	n=16	n=11	0.004
	4.2 (3.5, 6.0)	5.7 (4.1, 6.1)	3.5 (1.4, 4.0)	
Thalamus, Lactate	n=27	n=16	n=11	<0.001
	0.5 (0.3, 1.3)	0.4 (0.2, 0.5)	1.5 (0.6, 4.7)	
**Region of interest, Metabolite ratio, Median (IQR)**				
Basal ganglia, Lactate: N-acetyl-aspartate ratio	N=30	N=18	N=12	
	0.12 (0.05, 0.27)	0.07 (0.02, 0.14)	0.32 (0.11, 4.08)	0.01
Parietal-occipital gray matter, Lactate: N-acetyl-aspartate ratio	N=32	N=20	N=12	
	0.11 (0.06, 1.07)	0.07 (0.05, 0.13)	2.64 (0.11, 7.42)	0.003
Parietal-occipital white matter, Lactate: N-acetyl-aspartate ratio	N=29	N=17	N=12	
	0.20 (0.06, 0.73)	0.07 (0.04, 0.23)	0.85 (0.26, 15.46)	0.002
Thalamus, Lactate: N-acetyl-aspartate ratio	N=27	N=16	N=11	
	0.08 (0.05, 0.43)	0.06 (0.04, 0.08)	0.43 (0.15, 3.26)	0.001

Favorable vs. unfavorable groups compared using Kruskal-Wallis test

© 2023 Fink EL et al. *JAMA Network Open.*

eTable 5. Univariate and Stepwise, Multivariable Logistic Regressions for the Association Between Magnetic Resonance Imaging (MRI) And 1 Year Outcome. Stepwise selection with entry and stay level of 0.20. Multivariable area under the receiver operator curves (AUROC) were also calculated.

Variable	Univariate Odds Ratio (95% Wald CI	Univariate AUROC (95% CI)	Multivariable Odds Ratio (95% Wald CI	Multivariable AUROC with MRI (95% CI)	Multivariable AUROC without MRI (95% CI)	p-value*
**MRI Injury Score**[1]	1.11 (1.04, 1.17)	0.823 (0.712, 0.933)	1.12 (1.04, 1.20)	0.886 (0.786, 0.985)	0.755 (0.623, 0.888)	0.03
Age	0.96 (0.86, 1.06)	0.548 (0.411, 0.686)	0.91 (0.78, 1.07)			
Female sex	1.23 (0.44, 3.45)	0.525 (0.399, 0.652)	0.72 (0.17, 3.09)			
Witnessed status	0.08 (0.02, 0.30)	0.736 (0.624, 0.849)	0.05 (0.01, 0.30)			
**T2-weighted imaging**						
T2 Total score	1.13 (1.03, 1.25)	0.719 (0.588, 0.851)	1.19 (1.05, 1.36)	0.855 (0.750, 0.960)	0.755 (0.623, 0.888)	0.07

© 2023 Fink EL et al. *JAMA Network Open.*

Age	0.96 (0.86, 1.06)	0.548 (0.411, 0.686)	0.88 (0.75, 1.04)			
Female sex	1.23 (0.44, 3.45)	0.525 (0.399, 0.652)	0.74 (0.19, 2.91)			
Witnessed status	0.08 (0.02, 0.30)	0.736 (0.624, 0.849)	0.04 (0.01, 0.24)			
Cortex score[2]	1.13 (1.00, 1.27)	0.609 (0.473, 0.744)	1.18 (0.98, 1.41)	0.811 (0.695, 0.928)	0.755 (0.623, 0.888)	0.24
Age	0.96 (0.86, 1.06)	0.548 (0.411, 0.686)	0.90 (0.78, 1.04)			
Female sex	1.23 (0.44, 3.45)	0.525 (0.399, 0.652)	0.69 (0.18, 2.61)			
Witnessed status	0.08 (0.02, 0.30)	0.736 (0.624, 0.849)	0.05 (0.01, 0.25)			
Deep Gray Matter score[3]	1.61 (1.26, 2.06)	0.815 (0.705, 0.924)	1.70 (1.25, 2.31)	0.888 (0.793, 0.984)	0.755 (0.623, 0.888)	0.02

© 2023 Fink EL et al. *JAMA Network Open.*

Age	0.96 (0.86, 1.06)	0.548 (0.411, 0.686)	0.85 (0.70, 1.03)			
Female sex	1.23 (0.44, 3.45)	0.525 (0.399, 0.652)	1.01 (0.22, 4.61)			
Witnessed status	0.08 (0.02, 0.30)	0.736 (0.624, 0.849)	0.06 (0.01, 0.33)			
**Diffusion weighted imaging**						
DWI Total score	1.19 (1.08, 1.30)	0.842 (0.736, 0.949)	1.19 (1.07, 1.33)	0.887 (0.791, 0.983)	0.755 (0.623, 0.888)	0.02
Age	0.96 (0.86, 1.06)	0.548 (0.411, 0.686)	0.94 (0.81, 1.10)			
Female sex	1.23 (0.44, 3.45)	0.525 (0.399, 0.652)	0.77 (0.18, 3.30)			
Witnessed status	0.08 (0.02, 0.30)	0.736 (0.624, 0.849)	0.06 (0.01, 0.38)			

© 2023 Fink EL et al. *JAMA Network Open.*

Cortex score	1.28 (1.12, 1.48)	0.772 (0.642, 0.901)	1.31 (1.09, 1.57)	0.857 (0.740, 0.974)	0.755 (0.623, 0.888)	0.09
Age	0.96 (0.86, 1.06)	0.548 (0.411, 0.686)	0.94 (0.81, 1.09)			
Female sex	1.23 (0.44, 3.45)	0.525 (0.399, 0.652)	0.53 (0.12, 2.31)			
Witnessed status	0.08 (0.02, 0.30)	0.736 (0.624, 0.849)	0.06 (0.01, 0.36)			
Deep Gray Matter score	1.81 (1.26, 2.58)	0.824 (0.720, 0.928)	2.01 (1.32, 3.07)	0.900 (0.811, 0.988)	0.755 (0.623, 0.888)	0.013
Age	0.96 (0.86, 1.06)	0.548 (0.411, 0.686)	0.85 (0.71, 1.03)			
Female sex	1.23 (0.44, 3.45)	0.525 (0.399, 0.652)	1.16 (0.24, 5.57)			
Witnessed status	0.08 (0.02, 0.30)	0.736 (0.624, 0.849)	0.04 (0.01, 0.26)			

© 2023 Fink EL et al. *JAMA Network Open.*

* p-value comparing multivariable AUROC with and without MRI Injury Score

Variables initially tested in univariate models that had p>0.2: etiology, location, total number of epinephrine doses, first monitored rhythm

© 2023 Fink EL et al. *JAMA Network Open.*

eTable 6. Univariate Logistic Regression and Area Under The Receiver Operator Curves (AUROC)

Univariate logistic regression and area under the receiver operator curves (AUROC) were performed for magnetic resonance spectroscopy metabolites N-acetyl-aspartate and Lactate in all 4 regions of interest with 1 year outcome. Multivariable analyses were not conducted due to small sample size and resulting large confidence intervals.

**Metabolite and region of interest**	**Odds Ratio**	**95% Wald CI**	**AUROC**
**N-acetyl-aspartate**			
Basal ganglia	0.55	0.33, 0.93	0.764 (0.574, 0.954)
Occipital-parietal gray matter	0.54	0.34, 0.85	0.817 (0.655, 0.978)
Occipital-parietal white matter	0.59	0.37, 0.93	0.750 (0.552, 0.948)
Thalamus	0.39	0.19, 0.83	0.830 (0.665, 0.995)
**Lactate**			
Basal ganglia	9.35	0.85, 102.64	0.776 (0.582, 0.969)
Occipital-parietal gray matter	2.69	1.01, 7.15	0.783 (0.594, 0.973)
Occipital-parietal white matter	4.75	1.02, 22.05	0.858 (0.705, 1.000)
Thalamus	16.18	1.46, 178.99	0.898 (0.776, 1.000)

© 2023 Fink EL et al. *JAMA Network Open.*

eFigure 2. Representative Patient Brain Magnetic Resonance Imaging And Spectroscopy of Children With Cardiac Arrest With More Severe Impairment (A), Moderately Severe Impairment (B), and No Impairment (C) At 1 Year.

Patient A is a 14-mo white male with an unwitnessed out-of-hospital cardiac arrest due to asphyxia. Cardiopulmonary resuscitation was provided for 80 minutes, and his first rhythm was reported as sinus bradycardia. He survived with an unfavorable outcome at 1 year, with a VABS score of 40. Brain MRI T2-weighted imaging had injury in temporal gray matter, parietal and occipital white matter, basal ganglia, and thalamus. DWI showed injury in frontal and temporal gray and white matter, parietal and occipital white matter, the lenticular nucleus of the basal ganglia, and centrum semiovale. MRS had decreased NAA, and increased lactate and Lac:NAA ratio.

Patient B is a 12-mo black female with witnessed in-hospital cardiac arrest due to asphyxia presenting with sinus bradycardia as the first monitored rhythm. History of congenital heart disease and pulmonary disease. She had 2 minutes of cardiopulmonary resuscitation and had a favorable outcome at 1 year, scoring 88 on the VABS. Brain MRI had no acute lesions and brain MRS demonstrated moderate injury with decreased NAA.

Patient C is a 13-yo white female with witnessed out-of-hospital cardiac arrest due to cardiac etiology, having ventricular arrhythmia as the first monitored rhythm. She had 16 minutes of CPR and a favorable outcome at 1y, scoring 117 on the VABS. She had a normal MRI and MRS.

© 2023 Fink EL et al. *JAMA Network Open.*

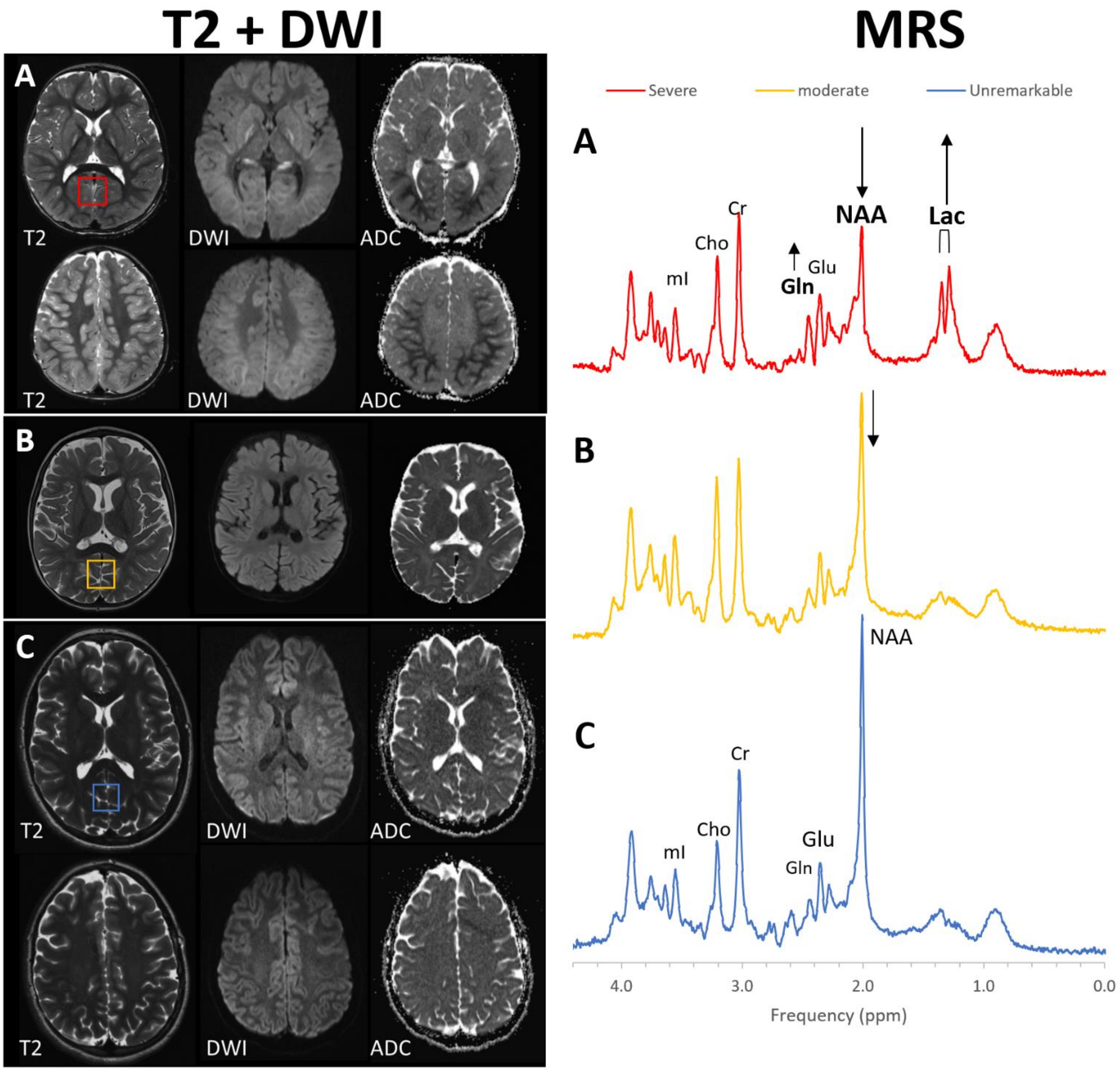

VABS, Vineland Adaptive Behavioral Scale; MRI, magnetic resonance imaging; i.u., institutional units; MRS, magnetic resonance spectroscopy; DWI, diffusion-weighted imaging; NAA, N-acetyl-aspartate; Lac, lactate

© 2023 Fink EL et al. *JAMA Network Open.*

eTable 7. Imaging Details for the Patients A, B, and C in Supplemental Figure 2.

Variable	Patient A	Patient B	Patient C
Days to brain MRI from the time of cardiac arrest	4	9	10
POCCA MRI Injury score	30	0	0
POCCA MRI T2 score	12	0	0
POCCA MRI DWI score	18	0	0
Occipital-parietal gray matter, N-acetyl-aspartate, i.u.	2.0	4.8	6.6
Occipital-parietal gray matter, Lactate, i.u.	3.5	0.3	0.3
Occipital-parietal gray matter, Lactate:N-acetyl-aspartate	1.76	0.06	0.05

© 2023 Fink EL et al. *JAMA Network Open.*

© 2023 Fink EL et al. *JAMA Network Open.*
