# Supplementary material for: Assessment of Brain Magnetic Resonance and Spectroscopy Imaging Findings and Outcomes After Pediatric Cardiac Arrest
Source: JAMA Netw Open. 2023 Jun 30;6(6):e2320713. doi: 10.1001/jamanetworkopen.2023.20713 (PMC10314315; doi:10.1001/jamanetworkopen.2023.20713)
Supplement: Supplement 3. — Data Sharing Statement [file jamanetwopen-e2320713-s003.pdf]

Data Sharing Statement

Fink. Assessment of Brain Magnetic Resonance and Spectroscopy Imaging Findings and Outcomes After Pediatric Cardiac Arrest. *JAMA Netw Open*. Published June 30, 2023. doi:10.1001/jamanetworkopen.2023.20713

Data
Data available: Yes
Data types: Data dictionary
How to access data: finkel@ccm.upmc.edu
When available: With publication

Supporting Documents
Document types: None

Additional Information
Who can access the data: Researchers whose proposed use of the data has been approved
Types of analyses: Specified purpose
Mechanisms of data availability: Signed data access agreement
